# Retraction Note: Down-regulated microRNA-124 expression as predictive biomarker and its prognostic significance with clinicopathological features in breast cancer patients

**DOI:** 10.1186/s13000-016-0560-9

**Published:** 2016-11-02

**Authors:** Ali Arabkheradmand, Aghdas Safari, Mehri Seifoleslami, Emad Yahaghi, Masoumeh Gity

**Affiliations:** 1 Department of Surgery, Cancer and Reconstructive Surgeon, Cancer Institute, School of Medicine, Tehran University of Medical Sciences, Tehran, Iran; 2 Department of Gynecology, Khanevadeh Hospital, AJA University of Medical Sciences, Tehran, Iran; 3 Department of Molecular Biology, Baqiyatallah University of Medical Sciences, Tehran, Iran; 4 Department of Radiology, Medical Imaging Center, Tehran University of Medical Sciences, Tehran, Iran

## Retraction

The Editor-in-Chief and Publisher have retracted this article [1] because the scientific integrity of the content cannot be guaranteed. An investigation by the Publisher found it to be one of a group of articles we have identified as showing evidence suggestive of attempts to subvert the peer review and publication system to inappropriately obtain or allocate authorship. This article showed evidence of plagiarism (most notably from the articles cited [2–4]) and authorship manipulation.
